# (μ-Acetato-κ^2^ *O*:*O*′)[μ-2,6-bis­({bis­[(pyri­din-2-yl-κ*N*)meth­yl]amino-κ*N*}meth­yl)-4-methyl­phenolato-κ^2^ *O*:*O*](metha­nol-κ*O*)dizinc bis­(perchlorate)

**DOI:** 10.1107/S1600536814004279

**Published:** 2014-03-05

**Authors:** Biswanath Das, Matti Haukka, Ebbe Nordlander

**Affiliations:** a Inorganic Chemistry Research Group, Chemical Physics, Center for Chemistry and Chemical Engineering, Lund University, Box 124, SE-221 00 Lund, Sweden; b Department of Chemistry, University of Jyvaskyla, PO Box 35, FI-40014 Jyväskylä, Finland

## Abstract

The binuclear title complex, [Zn_2_(C_33_H_33_N_6_O)(CH_3_COO_2_)(CH_3_OH)](ClO_4_)_2_, was synthesized by the reaction between 2,6-bis­({[bis­(pyridin-2-yl)meth­yl]amino}­meth­yl)-4-methyl­phenol (H-BPMP), Zn(OAc)_2_ and NaClO_4_. The two Zn^II^ ions are bridged by the phenolate O atom of the octadentate ligand and the acetate group. An additional methanol ligand is terminally coordinated to one of the Zn^II^ ions, rendering the whole structure unsymmetric. Other symmetric dizinc complexes of BPMP have been reported. However, to the best of our knowledge, the present structure, in which the two Zn^II^ ions are distinguishable by the number of coordinating ligands and the coordination geometries (octahedral and square-pyramidal), is unique. The dizinc complex is a dication, and two perchlorate anions balance the charge. The –OH group of the coordinating methanol solvent mol­ecule forms a hydrogen bond with a perchlorate counter-anion. One of the anions is disordered over two sets of sites with an occupancy ratio of 0.734 (2):0.266 (2).

## Related literature   

For the ligand synthesis and related dizinc complexes of the HBPMP ligand, see: Selmeczi *et al.* (2007 ▶); Torelli *et al.* (2000 ▶).
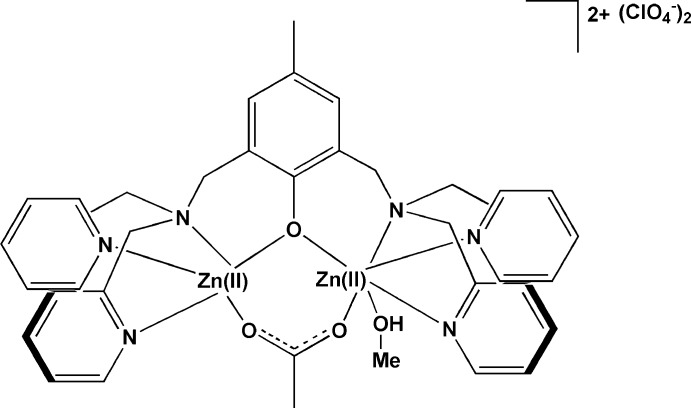


## Experimental   

### 

#### Crystal data  

[Zn_2_(C_33_H_33_N_6_O)(C_2_H_3_O_2_)(CH_4_O)](ClO_4_)_2_
*M* *_r_* = 950.38
Triclinic, 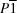

*a* = 10.0543 (4) Å
*b* = 10.7342 (4) Å
*c* = 18.7836 (7) Å
α = 86.320 (2)°
β = 80.372 (2)°
γ = 78.185 (2)°
*V* = 1955.38 (13) Å^3^
*Z* = 2
Mo *K*α radiation
μ = 1.43 mm^−1^
*T* = 100 K
0.35 × 0.33 × 0.18 mm

#### Data collection  

Bruker Kappa APEXII DUO CCD diffractometer
Absorption correction: multi-scan (*SADABS*; Sheldrick, 2008*a* ▶) *T*_min_ = 0.633, *T*_max_ = 0.786
62201 measured reflections
19190 independent reflections
15523 reflections with *I* > 2σ(*I*)
*R*_int_ = 0.016

#### Refinement  

*R*[*F* ^2^ > 2σ(*F* ^2^)] = 0.032
*wR*(*F* ^2^) = 0.087
*S* = 1.03
19190 reflections
563 parameters
H-atom parameters constrained
Δρ_max_ = 0.95 e Å^−3^
Δρ_min_ = −0.65 e Å^−3^

### 

Data collection: *APEX2* (Bruker, 2010 ▶); cell refinement: *SAINT* (Bruker, 2010 ▶); data reduction: *SAINT*; program(s) used to solve structure: *SHELXS97* (Sheldrick, 2008*b* ▶); program(s) used to refine structure: *SHELXL97* (Sheldrick, 2008*b* ▶); molecular graphics: *CrystalMaker* (CrystalMaker, 2011 ▶); software used to prepare material for publication: *SHELXL97*.

## Supplementary Material

Crystal structure: contains datablock(s) I, global. DOI: 10.1107/S1600536814004279/kj2234sup1.cif

Structure factors: contains datablock(s) I. DOI: 10.1107/S1600536814004279/kj2234Isup2.hkl

Supporting information file. DOI: 10.1107/S1600536814004279/kj2234Isup4.cdx

CCDC reference: 988598

Additional supporting information:  crystallographic information; 3D view; checkCIF report

## Figures and Tables

**Table 1:** Selected bond lengths (Å)

Zn1—O1	2.0574 (8)
Zn1—O2	2.0849 (8)
Zn1—O3	2.1013 (9)
Zn1—N2	2.1470 (10)
Zn1—N3	2.1524 (9)
Zn1—N1	2.1699 (9)
Zn2—O1	1.9932 (8)
Zn2—O4	2.0009 (8)
Zn2—N5	2.0747 (10)
Zn2—N6	2.1117 (10)
Zn2—N4	2.2025 (9)

**Table 2:** Hydrogen-bond geometry (Å, °)

*D*—H⋯*A*	*D*—H	H⋯*A*	*D*⋯*A*	*D*—H⋯*A*
O3—H3*O*⋯O5^i^	0.85	1.86	2.7022 (14)	170

Symmetry code: (i) 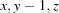.

**Fig. 1.**
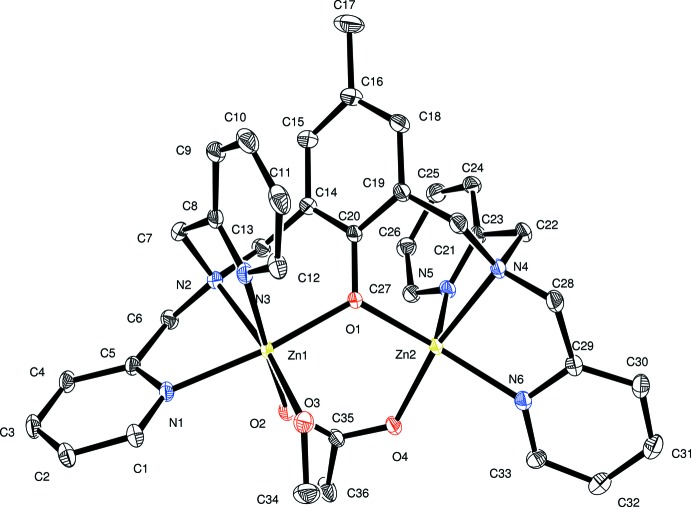
Plot of the molecular cation [Zn2(µ-OAc)(MeOH)(BPMP)]2+, showing 30% probability displacement ellipsoids. H atoms have been excluded for clarity.

## supplementary crystallographic information

### 1. Comment

The crystal and molecular structure of the dinuclear Zn^II^ complex [Zn_2_(µ-OAc)(MeOH)(BPMP)]^2+^, where OAc = acetate and *H*-BPMP = 2,6-bis[bis(2-pyridylmethyl)aminomethyl]-4-methylphenol, has been determined. The complex is rendered asymmetric by the coordination of the methanol molecule. Thus, the two metal sites may be distinguished by their coordination geometries and the number of donor groups at each metal center. Zn1 is in an N_3_O_3_ coordination environment with slightly distorted octahedral geometry and an average metal–ligand bond length of 2.119 Å; whereas Zn2 is in distorted square pyramidal geometry with N_3_O_2_ coordination geometry and with an average bond length of 2.077 Å. The Zn1—Zn2 distance is 3.5528 (2) Å and the two metals are bridged by the phenolate oxygen (O1) and the *syn*,*syn*-µ-1,3-acetate. The Zn1—O1—Zn2 angle is 122.57 (9)°, which is a value that is intermediate between those reported by Selmeczi *et al.* for the two dizinc complexes [Zn_2_(BPMP)(µ-OH)](ClO_4_)_2_ and [Zn_2_(BPMP)(H_2_O)_2_](ClO_4_)_3_ [96.04 (2) and 137.21(3, respectively; Selmeczi *et al.*, 2007]. A difference of 0.0840 (11) Å has been observed between the two Zn—O (acetate) bonds. The –OH group of the coordinated methanol solvent molecule forms an isolated hydrogen bond with a perhclorate counteranion.

### 2. Experimental

The ligand HBPMP was prepared by following the procedure reported by Torelli *et al.* (2000). For the synthesis of [Zn_2_(µ-acetato)(MeOH)(BPMP)](ClO_4_)_2_, a 30 ml methanolic solution of 0.25 g (0.471 mmol) of HBPMP in a 100 ml round bottom flask was prepared. To this solution, 0.173 g (0.943 mmol) of Zn(OAc)_2_ was added, and the solution was stirred for two hrs, followed by addition of 0.1153 g (0.942 mmol) of sodium perchlorate. The resultant solution was stirred vigorously for 1 hr. The solvent was removed under vacuum and washed initially with 10 ml of ice cold water to remove unreacted salts and thereafter with 20 ml of diethyl ether. The resultant solid was collected in a round bottom flask and was dried under vacuum to yield a white powder that was dissolved in 2 ml of dry methanol. Colorless crystals of [Zn_2_(µ-OAc)(MeOH)(BPMP)](ClO_4_)_2_ suitable for X-ray crystallography were grown from this methanol solution by slow diffusion of diethyl ether.

### 3. Refinement

The oxygen atoms of one of the ClO_4_^-^ anions were disordered over two sites with occupancy ratio of 0.73/0.27. The OH hydrogen atom was located from the difference Fourier map but the isotropic refinement was not satisfactory. Therefore, the OH hydrogen atom was constrained to ride on its parent atom, with *U*_iso_ = 1.5 *U*_eq_(parent atom). Other hydrogen atoms were positioned geometrically and constrained to ride on their parent atoms, with C—H = 0.95–0.99 Å, and *U*_iso_ = 1.2–1.5 *U*_eq_(parent atom). The highest peak is located 0.71 Å from atom Zn1 and the deepest hole is located 0.51 Å from atom Cl1.

### Crystal data

**Table:**

[Zn_2_(C_33_H_33_N_6_O)(C_2_H_3_O_2_)(CH_4_O)](ClO_4_)_2_	*Z* = 2
*M**_r_* = 950.38	*F*(000) = 976
Triclinic, *P*1	*D*_x_ = 1.614 Mg m^−^^3^
Hall symbol: -P 1	Mo *K*α radiation, λ = 0.71073 Å
*a* = 10.0543 (4) Å	Cell parameters from 9729 reflections
*b* = 10.7342 (4) Å	θ = 2.8–36.4°
*c* = 18.7836 (7) Å	µ = 1.43 mm^−^^1^
α = 86.320 (2)°	*T* = 100 K
β = 80.372 (2)°	Block, yellow
γ = 78.185 (2)°	0.35 × 0.33 × 0.18 mm
*V* = 1955.38 (13) Å^3^	

### Data collection

**Table:**

Bruker Kappa APEXII DUO CCD diffractometer	19190 independent reflections
Radiation source: fine-focus sealed tube	15523 reflections with *I* > 2σ(*I*)
Curved graphite crystal monochromator	*R*_int_ = 0.016
Detector resolution: 16 pixels mm^-1^	θ_max_ = 36.6°, θ_min_ = 2.1°
φ scans and ω scans with κ offset	*h* = −16→16
Absorption correction: multi-scan (*SADABS*; Sheldrick, 2008*a*)	*k* = −17→17
*T*_min_ = 0.633, *T*_max_ = 0.786	*l* = −30→31
62201 measured reflections	

### Refinement

**Table:**

Refinement on *F*^2^	Primary atom site location: structure-invariant direct methods
Least-squares matrix: full	Secondary atom site location: difference Fourier map
*R*[*F*^2^ > 2σ(*F*^2^)] = 0.032	Hydrogen site location: inferred from neighbouring sites
*wR*(*F*^2^) = 0.087	H-atom parameters constrained
*S* = 1.03	*w* = 1/[σ^2^(*F*_o_^2^) + (0.0437*P*)^2^ + 0.735*P*] where *P* = (*F*_o_^2^ + 2*F*_c_^2^)/3
19190 reflections	(Δ/σ)_max_ = 0.002
563 parameters	Δρ_max_ = 0.95 e Å^−^^3^
0 restraints	Δρ_min_ = −0.65 e Å^−^^3^

### Special details

**Table:**

Geometry. All e.s.d.'s (except the e.s.d. in the dihedral angle between two l.s. planes) are estimated using the full covariance matrix. The cell e.s.d.'s are taken into account individually in the estimation of e.s.d.'s in distances, angles and torsion angles; correlations between e.s.d.'s in cell parameters are only used when they are defined by crystal symmetry. An approximate (isotropic) treatment of cell e.s.d.'s is used for estimating e.s.d.'s involving l.s. planes.
Refinement. Refinement of *F*^2^ against ALL reflections. The weighted *R*-factor *wR* and goodness of fit *S* are based on *F*^2^, conventional *R*-factors *R* are based on *F*, with *F* set to zero for negative *F*^2^. The threshold expression of *F*^2^ > σ(*F*^2^) is used only for calculating *R*-factors(gt) *etc*. and is not relevant to the choice of reflections for refinement. *R*-factors based on *F*^2^ are statistically about twice as large as those based on *F*, and *R*- factors based on ALL data will be even larger.

### Fractional atomic coordinates and isotropic or equivalent isotropic displacement parameters (Å^2^)

**Table:**

	*x*	*y*	*z*	*U*_iso_*/*U*_eq_	Occ. (<1)
Zn1	0.404914 (12)	0.287963 (11)	0.196393 (7)	0.01438 (3)	
Zn2	0.762399 (12)	0.192005 (12)	0.203424 (7)	0.01622 (3)	
Cl1	0.64852 (3)	0.81110 (3)	0.138938 (18)	0.02784 (6)	
Cl2	0.04630 (4)	0.73238 (3)	0.416697 (16)	0.02788 (6)	
O1	0.56730 (8)	0.22892 (8)	0.25227 (4)	0.01726 (13)	
O2	0.51803 (8)	0.39983 (8)	0.12574 (5)	0.02011 (15)	
O3	0.45462 (10)	0.13837 (8)	0.12373 (5)	0.02328 (16)	
H3O	0.5090	0.0747	0.1396	0.035*	
O4	0.73783 (9)	0.29498 (9)	0.11224 (5)	0.02349 (16)	
O5	0.64811 (15)	0.93993 (11)	0.15863 (9)	0.0514 (4)	
O6	0.51205 (14)	0.80215 (14)	0.13164 (9)	0.0527 (3)	
O7	0.73777 (15)	0.78473 (14)	0.07170 (7)	0.0489 (3)	
O8	0.69998 (15)	0.72519 (12)	0.19353 (7)	0.0466 (3)	
O9	0.13246 (16)	0.74430 (14)	0.34787 (7)	0.0321 (3)	0.734 (2)
O10	0.0887 (3)	0.61728 (15)	0.45314 (10)	0.0500 (6)	0.734 (2)
O11	0.0592 (2)	0.83897 (13)	0.46055 (8)	0.0342 (4)	0.734 (2)
O12	−0.09405 (19)	0.7509 (2)	0.40768 (10)	0.0472 (5)	0.734 (2)
O9B	0.0062 (5)	0.6900 (4)	0.3461 (2)	0.0367 (10)	0.266 (2)
O10B	0.1895 (5)	0.7003 (5)	0.4069 (4)	0.0510 (14)	0.266 (2)
O11B	−0.0021 (7)	0.6269 (5)	0.4675 (3)	0.0397 (12)	0.266 (2)
O12B	−0.0205 (5)	0.8437 (3)	0.4349 (2)	0.0334 (10)	0.266 (2)
N1	0.22879 (9)	0.38476 (9)	0.14793 (5)	0.01711 (15)	
N2	0.31404 (10)	0.43817 (9)	0.27006 (5)	0.01806 (16)	
N3	0.28959 (10)	0.18295 (9)	0.27692 (5)	0.01951 (17)	
N4	0.81362 (10)	0.05214 (9)	0.29061 (5)	0.01938 (16)	
N5	0.82889 (10)	0.31006 (9)	0.26733 (5)	0.01902 (16)	
N6	0.92968 (10)	0.06431 (9)	0.14755 (5)	0.01880 (16)	
C1	0.16155 (11)	0.33903 (11)	0.10315 (7)	0.02043 (19)	
H1	0.1965	0.2554	0.0860	0.025*	
C2	0.04273 (12)	0.40860 (12)	0.08061 (7)	0.0232 (2)	
H2	−0.0027	0.3736	0.0486	0.028*	
C3	−0.00807 (12)	0.53026 (12)	0.10589 (7)	0.0238 (2)	
H3	−0.0888	0.5805	0.0912	0.029*	
C4	0.06054 (12)	0.57767 (11)	0.15289 (7)	0.0225 (2)	
H4	0.0267	0.6605	0.1713	0.027*	
C5	0.17950 (11)	0.50276 (10)	0.17279 (6)	0.01827 (18)	
C6	0.25960 (12)	0.54702 (10)	0.22410 (6)	0.02066 (19)	
H6A	0.3365	0.5830	0.1964	0.025*	
H6B	0.1989	0.6144	0.2547	0.025*	
C7	0.20189 (12)	0.39622 (12)	0.32153 (6)	0.0219 (2)	
H7A	0.1143	0.4227	0.3022	0.026*	
H7B	0.1915	0.4384	0.3680	0.026*	
C8	0.23019 (11)	0.25369 (12)	0.33441 (6)	0.0208 (2)	
C9	0.19014 (12)	0.19933 (14)	0.40185 (7)	0.0269 (2)	
H9	0.1503	0.2514	0.4420	0.032*	
C10	0.20931 (14)	0.06843 (15)	0.40935 (8)	0.0313 (3)	
H10	0.1816	0.0291	0.4546	0.038*	
C11	0.26965 (14)	−0.00504 (13)	0.34994 (8)	0.0306 (3)	
H11	0.2831	−0.0953	0.3538	0.037*	
C12	0.30993 (12)	0.05536 (11)	0.28486 (7)	0.0239 (2)	
H12	0.3533	0.0050	0.2445	0.029*	
C13	0.42082 (12)	0.46870 (11)	0.30836 (6)	0.02080 (19)	
H13A	0.3809	0.5439	0.3383	0.025*	
H13B	0.4976	0.4900	0.2725	0.025*	
C14	0.47481 (12)	0.35782 (11)	0.35572 (6)	0.01978 (18)	
C15	0.45211 (14)	0.36787 (13)	0.43074 (7)	0.0250 (2)	
H15	0.4026	0.4458	0.4513	0.030*	
C16	0.49986 (14)	0.26715 (14)	0.47628 (7)	0.0279 (2)	
C17	0.4717 (2)	0.27899 (19)	0.55740 (8)	0.0450 (4)	
H17A	0.4676	0.3673	0.5694	0.068*	
H17B	0.5455	0.2229	0.5788	0.068*	
H17C	0.3836	0.2544	0.5766	0.068*	
C18	0.57440 (13)	0.15521 (13)	0.44460 (7)	0.0249 (2)	
H18	0.6094	0.0859	0.4747	0.030*	
C19	0.59933 (11)	0.14172 (11)	0.36993 (6)	0.02006 (19)	
C20	0.54680 (11)	0.24273 (11)	0.32473 (6)	0.01771 (17)	
C21	0.68633 (12)	0.02336 (11)	0.33656 (7)	0.0219 (2)	
H21A	0.6330	−0.0138	0.3066	0.026*	
H21B	0.7121	−0.0399	0.3751	0.026*	
C22	0.89557 (12)	0.11097 (12)	0.33267 (6)	0.0218 (2)	
H22A	0.9944	0.0843	0.3131	0.026*	
H22B	0.8812	0.0793	0.3835	0.026*	
C23	0.85776 (11)	0.25427 (11)	0.33065 (6)	0.01980 (19)	
C24	0.85952 (12)	0.32543 (13)	0.38967 (7)	0.0234 (2)	
H24	0.8776	0.2843	0.4343	0.028*	
C25	0.83471 (13)	0.45668 (14)	0.38262 (7)	0.0260 (2)	
H25	0.8378	0.5069	0.4219	0.031*	
C26	0.80504 (13)	0.51447 (12)	0.31695 (7)	0.0249 (2)	
H26	0.7881	0.6045	0.3106	0.030*	
C27	0.80086 (12)	0.43792 (11)	0.26140 (6)	0.02130 (19)	
H27	0.7772	0.4771	0.2172	0.026*	
C28	0.89733 (13)	−0.06454 (11)	0.25708 (7)	0.0235 (2)	
H28A	0.8362	−0.1196	0.2457	0.028*	
H28B	0.9555	−0.1123	0.2913	0.028*	
C29	0.98728 (12)	−0.03182 (11)	0.18882 (6)	0.02039 (19)	
C30	1.12006 (13)	−0.09959 (12)	0.16848 (7)	0.0246 (2)	
H30	1.1608	−0.1638	0.1998	0.029*	
C31	1.19249 (14)	−0.07226 (13)	0.10175 (8)	0.0281 (2)	
H31	1.2837	−0.1173	0.0868	0.034*	
C32	1.13011 (14)	0.02149 (12)	0.05717 (7)	0.0264 (2)	
H32	1.1759	0.0395	0.0103	0.032*	
C33	0.99908 (12)	0.08864 (11)	0.08247 (6)	0.02099 (19)	
H33	0.9569	0.1545	0.0525	0.025*	
C34	0.49460 (18)	0.15787 (14)	0.04811 (7)	0.0329 (3)	
H34A	0.4616	0.0974	0.0217	0.049*	
H34B	0.5952	0.1445	0.0368	0.049*	
H34C	0.4545	0.2451	0.0338	0.049*	
C39	0.64042 (11)	0.38137 (11)	0.09515 (6)	0.01909 (18)	
C50	0.67620 (14)	0.46809 (15)	0.03151 (8)	0.0331 (3)	
H50A	0.6221	0.4602	−0.0062	0.050*	
H50B	0.7745	0.4441	0.0124	0.050*	
H50C	0.6552	0.5564	0.0470	0.050*	

### Atomic displacement parameters (Å^2^)

**Table:**

	*U*^11^	*U*^22^	*U*^33^	*U*^12^	*U*^13^	*U*^23^
Zn1	0.01089 (5)	0.01544 (5)	0.01542 (5)	0.00063 (4)	−0.00265 (4)	0.00095 (4)
Zn2	0.01181 (5)	0.02042 (6)	0.01527 (5)	−0.00020 (4)	−0.00364 (4)	0.00250 (4)
Cl1	0.02812 (14)	0.02265 (12)	0.02927 (14)	0.00642 (10)	−0.00913 (11)	0.00039 (10)
Cl2	0.03615 (16)	0.02869 (13)	0.01692 (11)	−0.00313 (11)	−0.00466 (11)	0.00407 (9)
O1	0.0122 (3)	0.0231 (3)	0.0150 (3)	0.0000 (3)	−0.0029 (2)	0.0012 (3)
O2	0.0152 (3)	0.0223 (3)	0.0212 (4)	−0.0020 (3)	−0.0026 (3)	0.0047 (3)
O3	0.0256 (4)	0.0178 (3)	0.0237 (4)	0.0036 (3)	−0.0049 (3)	−0.0022 (3)
O4	0.0160 (3)	0.0325 (4)	0.0185 (4)	0.0012 (3)	−0.0033 (3)	0.0075 (3)
O5	0.0559 (8)	0.0244 (5)	0.0764 (10)	0.0123 (5)	−0.0390 (7)	−0.0103 (5)
O6	0.0332 (6)	0.0538 (8)	0.0721 (10)	−0.0047 (6)	−0.0165 (6)	−0.0005 (7)
O7	0.0481 (7)	0.0544 (8)	0.0323 (6)	0.0102 (6)	0.0007 (5)	0.0030 (5)
O8	0.0610 (8)	0.0367 (6)	0.0358 (6)	0.0086 (5)	−0.0166 (6)	0.0106 (5)
O9	0.0359 (7)	0.0380 (7)	0.0181 (6)	−0.0001 (6)	0.0015 (5)	−0.0060 (5)
O10	0.1041 (19)	0.0174 (6)	0.0296 (8)	−0.0013 (9)	−0.0282 (11)	0.0031 (5)
O11	0.0595 (11)	0.0219 (6)	0.0210 (6)	−0.0149 (6)	0.0042 (6)	−0.0056 (4)
O12	0.0377 (9)	0.0678 (12)	0.0394 (9)	−0.0215 (8)	−0.0073 (7)	0.0125 (8)
O9B	0.059 (3)	0.0264 (17)	0.0257 (18)	−0.0033 (17)	−0.0166 (18)	0.0019 (13)
O10B	0.032 (2)	0.038 (2)	0.084 (4)	−0.0089 (18)	−0.007 (2)	−0.006 (2)
O11B	0.066 (3)	0.032 (2)	0.028 (2)	−0.021 (2)	−0.014 (2)	0.0086 (16)
O12B	0.050 (3)	0.0197 (15)	0.0257 (18)	−0.0028 (15)	0.0020 (17)	−0.0029 (12)
N1	0.0125 (3)	0.0172 (3)	0.0203 (4)	0.0002 (3)	−0.0035 (3)	0.0016 (3)
N2	0.0157 (4)	0.0191 (4)	0.0172 (4)	0.0012 (3)	−0.0026 (3)	0.0005 (3)
N3	0.0127 (4)	0.0219 (4)	0.0229 (4)	−0.0021 (3)	−0.0036 (3)	0.0046 (3)
N4	0.0144 (4)	0.0223 (4)	0.0198 (4)	−0.0001 (3)	−0.0044 (3)	0.0038 (3)
N5	0.0142 (4)	0.0249 (4)	0.0182 (4)	−0.0041 (3)	−0.0041 (3)	0.0026 (3)
N6	0.0159 (4)	0.0202 (4)	0.0200 (4)	−0.0023 (3)	−0.0040 (3)	0.0001 (3)
C1	0.0157 (4)	0.0196 (4)	0.0264 (5)	−0.0025 (3)	−0.0070 (4)	0.0026 (4)
C2	0.0166 (4)	0.0256 (5)	0.0289 (6)	−0.0051 (4)	−0.0095 (4)	0.0061 (4)
C3	0.0136 (4)	0.0259 (5)	0.0296 (6)	0.0007 (4)	−0.0058 (4)	0.0074 (4)
C4	0.0171 (4)	0.0200 (4)	0.0265 (5)	0.0039 (4)	−0.0028 (4)	0.0028 (4)
C5	0.0145 (4)	0.0179 (4)	0.0199 (5)	0.0013 (3)	−0.0019 (3)	0.0019 (3)
C6	0.0223 (5)	0.0162 (4)	0.0215 (5)	0.0027 (4)	−0.0058 (4)	−0.0009 (3)
C7	0.0158 (4)	0.0266 (5)	0.0193 (5)	0.0020 (4)	0.0006 (4)	0.0011 (4)
C8	0.0119 (4)	0.0283 (5)	0.0207 (5)	−0.0020 (4)	−0.0027 (3)	0.0052 (4)
C9	0.0160 (5)	0.0410 (7)	0.0219 (5)	−0.0047 (4)	−0.0033 (4)	0.0095 (5)
C10	0.0213 (5)	0.0421 (7)	0.0305 (6)	−0.0091 (5)	−0.0080 (5)	0.0183 (5)
C11	0.0222 (5)	0.0297 (6)	0.0407 (7)	−0.0077 (4)	−0.0104 (5)	0.0166 (5)
C12	0.0177 (5)	0.0225 (5)	0.0318 (6)	−0.0044 (4)	−0.0072 (4)	0.0073 (4)
C13	0.0222 (5)	0.0212 (4)	0.0192 (5)	−0.0021 (4)	−0.0060 (4)	−0.0019 (4)
C14	0.0177 (4)	0.0248 (5)	0.0164 (4)	−0.0024 (4)	−0.0039 (3)	0.0002 (4)
C15	0.0252 (5)	0.0313 (6)	0.0177 (5)	−0.0029 (4)	−0.0040 (4)	−0.0019 (4)
C16	0.0281 (6)	0.0382 (6)	0.0165 (5)	−0.0047 (5)	−0.0046 (4)	0.0022 (4)
C17	0.0611 (11)	0.0512 (9)	0.0171 (6)	0.0012 (8)	−0.0056 (6)	0.0007 (6)
C18	0.0221 (5)	0.0335 (6)	0.0183 (5)	−0.0041 (4)	−0.0054 (4)	0.0066 (4)
C19	0.0153 (4)	0.0257 (5)	0.0185 (5)	−0.0035 (4)	−0.0037 (3)	0.0049 (4)
C20	0.0126 (4)	0.0242 (4)	0.0158 (4)	−0.0025 (3)	−0.0032 (3)	0.0025 (3)
C21	0.0177 (5)	0.0243 (5)	0.0223 (5)	−0.0026 (4)	−0.0036 (4)	0.0070 (4)
C22	0.0157 (4)	0.0290 (5)	0.0202 (5)	−0.0006 (4)	−0.0077 (4)	0.0036 (4)
C23	0.0127 (4)	0.0284 (5)	0.0185 (4)	−0.0036 (4)	−0.0048 (3)	0.0030 (4)
C24	0.0172 (5)	0.0351 (6)	0.0189 (5)	−0.0055 (4)	−0.0055 (4)	0.0004 (4)
C25	0.0214 (5)	0.0355 (6)	0.0228 (5)	−0.0080 (5)	−0.0038 (4)	−0.0040 (4)
C26	0.0223 (5)	0.0273 (5)	0.0254 (5)	−0.0066 (4)	−0.0026 (4)	−0.0014 (4)
C27	0.0180 (5)	0.0252 (5)	0.0204 (5)	−0.0050 (4)	−0.0026 (4)	0.0027 (4)
C28	0.0206 (5)	0.0217 (5)	0.0251 (5)	0.0019 (4)	−0.0047 (4)	0.0050 (4)
C29	0.0173 (4)	0.0200 (4)	0.0235 (5)	−0.0012 (3)	−0.0056 (4)	−0.0001 (4)
C30	0.0189 (5)	0.0226 (5)	0.0307 (6)	0.0017 (4)	−0.0059 (4)	−0.0032 (4)
C31	0.0195 (5)	0.0283 (6)	0.0342 (7)	−0.0005 (4)	−0.0004 (5)	−0.0078 (5)
C32	0.0244 (5)	0.0265 (5)	0.0271 (6)	−0.0059 (4)	0.0023 (4)	−0.0055 (4)
C33	0.0221 (5)	0.0203 (4)	0.0207 (5)	−0.0047 (4)	−0.0021 (4)	−0.0025 (4)
C34	0.0425 (8)	0.0318 (6)	0.0216 (6)	0.0017 (6)	−0.0063 (5)	−0.0072 (5)
C39	0.0154 (4)	0.0246 (5)	0.0177 (4)	−0.0042 (4)	−0.0056 (3)	0.0051 (3)
C50	0.0190 (5)	0.0464 (8)	0.0323 (7)	−0.0078 (5)	−0.0065 (5)	0.0222 (6)

### Geometric parameters (Å, º)

**Table:**

Zn1—O1	2.0574 (8)	C8—C9	1.3921 (16)
Zn1—O2	2.0849 (8)	C9—C10	1.380 (2)
Zn1—O3	2.1013 (9)	C9—H9	0.9500
Zn1—N2	2.1470 (10)	C10—C11	1.388 (2)
Zn1—N3	2.1524 (9)	C10—H10	0.9500
Zn1—N1	2.1699 (9)	C11—C12	1.3852 (18)
Zn2—O1	1.9932 (8)	C11—H11	0.9500
Zn2—O4	2.0009 (8)	C12—H12	0.9500
Zn2—N5	2.0747 (10)	C13—C14	1.5045 (16)
Zn2—N6	2.1117 (10)	C13—H13A	0.9900
Zn2—N4	2.2025 (9)	C13—H13B	0.9900
Cl1—O6	1.4245 (14)	C14—C15	1.3969 (17)
Cl1—O8	1.4249 (11)	C14—C20	1.4051 (16)
Cl1—O7	1.4294 (13)	C15—C16	1.3919 (18)
Cl1—O5	1.4533 (12)	C15—H15	0.9500
Cl2—O12B	1.281 (4)	C16—C18	1.390 (2)
Cl2—O10B	1.394 (5)	C16—C17	1.511 (2)
Cl2—O10	1.3965 (15)	C17—H17A	0.9800
Cl2—O12	1.4214 (18)	C17—H17B	0.9800
Cl2—O9	1.4448 (14)	C17—H17C	0.9800
Cl2—O11	1.4906 (14)	C18—C19	1.3946 (17)
Cl2—O11B	1.536 (4)	C18—H18	0.9500
Cl2—O9B	1.568 (4)	C19—C20	1.4046 (15)
O1—C20	1.3552 (13)	C19—C21	1.4953 (17)
O2—C39	1.2497 (14)	C21—H21A	0.9900
O3—C34	1.4241 (17)	C21—H21B	0.9900
O3—H3O	0.8547	C22—C23	1.5067 (17)
O4—C39	1.2716 (13)	C22—H22A	0.9900
N1—C1	1.3359 (15)	C22—H22B	0.9900
N1—C5	1.3451 (14)	C23—C24	1.3905 (18)
N2—C6	1.4754 (14)	C24—C25	1.3812 (19)
N2—C7	1.4819 (15)	C24—H24	0.9500
N2—C13	1.4866 (15)	C25—C26	1.3944 (18)
N3—C8	1.3429 (16)	C25—H25	0.9500
N3—C12	1.3450 (15)	C26—C27	1.3800 (18)
N4—C28	1.4718 (16)	C26—H26	0.9500
N4—C22	1.4821 (16)	C27—H27	0.9500
N4—C21	1.4955 (15)	C28—C29	1.5066 (17)
N5—C27	1.3447 (15)	C28—H28A	0.9900
N5—C23	1.3455 (14)	C28—H28B	0.9900
N6—C33	1.3407 (15)	C29—C30	1.3873 (17)
N6—C29	1.3431 (14)	C30—C31	1.386 (2)
C1—C2	1.3883 (16)	C30—H30	0.9500
C1—H1	0.9500	C31—C32	1.384 (2)
C2—C3	1.3847 (18)	C31—H31	0.9500
C2—H2	0.9500	C32—C33	1.3881 (18)
C3—C4	1.3854 (19)	C32—H32	0.9500
C3—H3	0.9500	C33—H33	0.9500
C4—C5	1.3906 (15)	C34—H34A	0.9800
C4—H4	0.9500	C34—H34B	0.9800
C5—C6	1.5094 (17)	C34—H34C	0.9800
C6—H6A	0.9900	C39—C50	1.5084 (16)
C6—H6B	0.9900	C50—H50A	0.9800
C7—C8	1.5092 (17)	C50—H50B	0.9800
C7—H7A	0.9900	C50—H50C	0.9800
C7—H7B	0.9900		
			
O1—Zn1—O2	90.95 (3)	C8—C7—H7A	109.3
O1—Zn1—O3	97.15 (3)	N2—C7—H7B	109.3
O2—Zn1—O3	91.81 (4)	C8—C7—H7B	109.3
O1—Zn1—N2	91.74 (3)	H7A—C7—H7B	107.9
O2—Zn1—N2	94.53 (4)	N3—C8—C9	122.20 (12)
O3—Zn1—N2	169.00 (4)	N3—C8—C7	116.56 (10)
O1—Zn1—N3	86.70 (3)	C9—C8—C7	121.17 (12)
O2—Zn1—N3	174.90 (4)	C10—C9—C8	118.80 (13)
O3—Zn1—N3	92.97 (4)	C10—C9—H9	120.6
N2—Zn1—N3	81.04 (4)	C8—C9—H9	120.6
O1—Zn1—N1	168.87 (4)	C9—C10—C11	119.19 (12)
O2—Zn1—N1	86.08 (3)	C9—C10—H10	120.4
O3—Zn1—N1	93.66 (4)	C11—C10—H10	120.4
N2—Zn1—N1	77.83 (4)	C12—C11—C10	118.92 (12)
N3—Zn1—N1	95.38 (3)	C12—C11—H11	120.5
O1—Zn2—O4	98.18 (3)	C10—C11—H11	120.5
O1—Zn2—N5	94.83 (4)	N3—C12—C11	122.16 (13)
O4—Zn2—N5	104.35 (4)	N3—C12—H12	118.9
O1—Zn2—N6	149.41 (4)	C11—C12—H12	118.9
O4—Zn2—N6	90.61 (4)	N2—C13—C14	110.86 (9)
N5—Zn2—N6	111.34 (4)	N2—C13—H13A	109.5
O1—Zn2—N4	89.52 (3)	C14—C13—H13A	109.5
O4—Zn2—N4	169.51 (4)	N2—C13—H13B	109.5
N5—Zn2—N4	81.95 (4)	C14—C13—H13B	109.5
N6—Zn2—N4	79.21 (4)	H13A—C13—H13B	108.1
O6—Cl1—O8	111.94 (9)	C15—C14—C20	119.55 (10)
O6—Cl1—O7	109.80 (9)	C15—C14—C13	120.33 (11)
O8—Cl1—O7	109.16 (8)	C20—C14—C13	120.11 (10)
O6—Cl1—O5	108.75 (8)	C16—C15—C14	121.96 (12)
O8—Cl1—O5	108.31 (8)	C16—C15—H15	119.0
O7—Cl1—O5	108.83 (10)	C14—C15—H15	119.0
O12B—Cl2—O10B	122.5 (3)	C18—C16—C15	117.73 (11)
O12B—Cl2—O10	135.8 (2)	C18—C16—C17	121.11 (13)
O10B—Cl2—O10	69.9 (3)	C15—C16—C17	121.16 (13)
O12B—Cl2—O12	67.9 (3)	C16—C17—H17A	109.5
O10B—Cl2—O12	164.3 (3)	C16—C17—H17B	109.5
O10—Cl2—O12	111.87 (15)	H17A—C17—H17B	109.5
O12B—Cl2—O9	108.17 (18)	C16—C17—H17C	109.5
O10B—Cl2—O9	56.6 (3)	H17A—C17—H17C	109.5
O10—Cl2—O9	112.33 (13)	H17B—C17—H17C	109.5
O12—Cl2—O9	110.44 (10)	C16—C18—C19	121.93 (11)
O10B—Cl2—O11	86.4 (2)	C16—C18—H18	119.0
O10—Cl2—O11	108.75 (10)	C19—C18—H18	119.0
O12—Cl2—O11	107.04 (12)	C18—C19—C20	119.73 (11)
O9—Cl2—O11	106.09 (8)	C18—C19—C21	121.19 (10)
O12B—Cl2—O11B	112.4 (3)	C20—C19—C21	119.02 (10)
O10B—Cl2—O11B	104.9 (3)	O1—C20—C19	120.02 (10)
O12—Cl2—O11B	78.9 (3)	O1—C20—C14	120.94 (9)
O9—Cl2—O11B	138.7 (2)	C19—C20—C14	119.03 (10)
O11—Cl2—O11B	109.2 (2)	C19—C21—N4	110.63 (9)
O12B—Cl2—O9B	111.8 (3)	C19—C21—H21A	109.5
O10B—Cl2—O9B	104.8 (3)	N4—C21—H21A	109.5
O10—Cl2—O9B	103.47 (16)	C19—C21—H21B	109.5
O12—Cl2—O9B	59.5 (2)	N4—C21—H21B	109.5
O9—Cl2—O9B	59.87 (19)	H21A—C21—H21B	108.1
O11—Cl2—O9B	147.78 (15)	N4—C22—C23	112.64 (9)
O11B—Cl2—O9B	97.2 (2)	N4—C22—H22A	109.1
C20—O1—Zn2	116.39 (6)	C23—C22—H22A	109.1
C20—O1—Zn1	119.30 (6)	N4—C22—H22B	109.1
Zn2—O1—Zn1	122.58 (4)	C23—C22—H22B	109.1
C39—O2—Zn1	132.81 (7)	H22A—C22—H22B	107.8
C34—O3—Zn1	122.56 (8)	N5—C23—C24	121.68 (11)
C34—O3—H3O	110.3	N5—C23—C22	116.27 (10)
Zn1—O3—H3O	111.1	C24—C23—C22	121.97 (10)
C39—O4—Zn2	132.45 (8)	C25—C24—C23	119.24 (11)
C1—N1—C5	119.00 (9)	C25—C24—H24	120.4
C1—N1—Zn1	128.68 (7)	C23—C24—H24	120.4
C5—N1—Zn1	112.10 (7)	C24—C25—C26	119.05 (12)
C6—N2—C7	111.21 (9)	C24—C25—H25	120.5
C6—N2—C13	111.14 (9)	C26—C25—H25	120.5
C7—N2—C13	111.29 (9)	C27—C26—C25	118.52 (12)
C6—N2—Zn1	104.81 (7)	C27—C26—H26	120.7
C7—N2—Zn1	108.42 (7)	C25—C26—H26	120.7
C13—N2—Zn1	109.74 (7)	N5—C27—C26	122.62 (11)
C8—N3—C12	118.70 (10)	N5—C27—H27	118.7
C8—N3—Zn1	111.57 (7)	C26—C27—H27	118.7
C12—N3—Zn1	125.70 (8)	N4—C28—C29	110.33 (9)
C28—N4—C22	110.45 (9)	N4—C28—H28A	109.6
C28—N4—C21	110.18 (9)	C29—C28—H28A	109.6
C22—N4—C21	111.60 (9)	N4—C28—H28B	109.6
C28—N4—Zn2	107.91 (7)	C29—C28—H28B	109.6
C22—N4—Zn2	105.41 (7)	H28A—C28—H28B	108.1
C21—N4—Zn2	111.13 (7)	N6—C29—C30	121.89 (11)
C27—N5—C23	118.83 (11)	N6—C29—C28	115.99 (10)
C27—N5—Zn2	123.99 (8)	C30—C29—C28	122.08 (10)
C23—N5—Zn2	113.17 (8)	C31—C30—C29	119.06 (12)
C33—N6—C29	118.65 (10)	C31—C30—H30	120.5
C33—N6—Zn2	124.81 (8)	C29—C30—H30	120.5
C29—N6—Zn2	114.47 (8)	C32—C31—C30	119.09 (12)
N1—C1—C2	122.73 (11)	C32—C31—H31	120.5
N1—C1—H1	118.6	C30—C31—H31	120.5
C2—C1—H1	118.6	C31—C32—C33	118.48 (12)
C3—C2—C1	118.44 (11)	C31—C32—H32	120.8
C3—C2—H2	120.8	C33—C32—H32	120.8
C1—C2—H2	120.8	N6—C33—C32	122.62 (11)
C2—C3—C4	119.05 (10)	N6—C33—H33	118.7
C2—C3—H3	120.5	C32—C33—H33	118.7
C4—C3—H3	120.5	O3—C34—H34A	109.5
C3—C4—C5	119.31 (11)	O3—C34—H34B	109.5
C3—C4—H4	120.3	H34A—C34—H34B	109.5
C5—C4—H4	120.3	O3—C34—H34C	109.5
N1—C5—C4	121.46 (11)	H34A—C34—H34C	109.5
N1—C5—C6	115.97 (9)	H34B—C34—H34C	109.5
C4—C5—C6	122.57 (10)	O2—C39—O4	125.96 (10)
N2—C6—C5	109.33 (9)	O2—C39—C50	117.32 (10)
N2—C6—H6A	109.8	O4—C39—C50	116.71 (10)
C5—C6—H6A	109.8	C39—C50—H50A	109.5
N2—C6—H6B	109.8	C39—C50—H50B	109.5
C5—C6—H6B	109.8	H50A—C50—H50B	109.5
H6A—C6—H6B	108.3	C39—C50—H50C	109.5
N2—C7—C8	111.72 (9)	H50A—C50—H50C	109.5
N2—C7—H7A	109.3	H50B—C50—H50C	109.5

### Hydrogen-bond geometry (Å, º)

**Table:**

*D*—H···*A*	*D*—H	H···*A*	*D*···*A*	*D*—H···*A*
O3—H3*O*···O5^i^	0.85	1.86	2.7022 (14)	170

Symmetry code: (i) *x*, *y*−1, *z*.
